# Signal Transduction by VIP and PACAP Receptors

**DOI:** 10.3390/biomedicines10020406

**Published:** 2022-02-09

**Authors:** Ingrid Langer, Jérôme Jeandriens, Alain Couvineau, Swapnil Sanmukh, Dorota Latek

**Affiliations:** 1 Institut de Recherche Interdisciplinaire en Biologie Humaine et Moléculaire (IRIBHM), Université libre de Bruxelles, B-1070 Brussels, Belgium; Ingrid.Langer@ulb.be (I.L.); jerome.jeandriens@ulb.be (J.J.); 2 UMR 1149 Inserm, Centre de Recherche sur l’Inflammation (CRI), Université de Paris, 75018 Paris, France; alain.couvineau@inserm.fr; 3 Faculty of Chemistry, University of Warsaw, 02-093 Warsaw, Poland; swamukh1985in@gmail.com

**Keywords:** vasoactive intestinal polypeptide, pituitary adenylate cyclase activating polypeptide, neuropeptides, G protein-coupled receptors, VPAC1, VPAC2, PAC1, class B, secretin-like GPCRs, microswitches, receptor activation, splice variants, splicing, sequence conservation, gene co-occurrence

## Abstract

Homeostasis of the human immune system is regulated by many cellular components, including two neuropeptides, VIP and PACAP, primary stimuli for three class B G protein-coupled receptors, VPAC1, VPAC2, and PAC1. Vasoactive intestinal peptide (VIP) and pituitary adenylate cyclase-activating polypeptide (PACAP) regulate intestinal motility and secretion and influence the functioning of the endocrine and immune systems. Inhibition of VIP and PACAP receptors is an emerging concept for new pharmacotherapies for chronic inflammation and cancer, while activation of their receptors provides neuroprotection. A small number of known active compounds for these receptors still impose limitations on their use in therapeutics. Recent cryo-EM structures of VPAC1 and PAC1 receptors in their agonist-bound active state have provided insights regarding their mechanism of activation. Here, we describe major molecular switches of VPAC1, VPAC2, and PAC1 that may act as triggers for receptor activation and compare them with similar non-covalent interactions changing upon activation that were observed for other GPCRs. Interhelical interactions in VIP and PACAP receptors that are important for agonist binding and/or activation provide a molecular basis for the design of novel selective drugs demonstrating anti-inflammatory, anti-cancer, and neuroprotective effects. The impact of genetic variants of VIP, PACAP, and their receptors on signalling mediated by endogenous agonists is also described. This sequence diversity resulting from gene splicing has a significant impact on agonist selectivity and potency as well as on the signalling properties of VIP and PACAP receptors.

## 1. Introduction

VPAC1, VPAC2, and PAC1 receptors are secretin-like G protein-coupled receptors, constituting one of five main GPCR classes regulating responses to extracellular stimuli in humans. Secretin-like GPCRs (class B GPCRs) interact with peptide hormones, such as secretin, glucagon-like peptides (GLP-1 and GLP-2), growth hormone-releasing hormone (GHRH), pituitary adenylate cyclase-activating peptide (PACAP), vasoactive intestinal peptide (VIP), corticotropin-releasing hormone (CRH), parathyroid hormone (PTH), and calcitonin-related peptides, through their large extracellular N-terminal domain [1]. Like other GPCRs, class B GPCRs regulate intracellular concentrations of cAMP by coupling to adenylate cyclase (AC) through Gαs and through Gαi/Gαq-mediated activation of phospholipase C (PLC) [2].

Secretin-like GPCRs share the heptahelical transmembrane fold (7TM) with other GPCR classes due to function conservation rather than sequence conservation. Class B GPCRs share 20–45% sequence similarity within their class members but less then 10% with members of other GPCR classes [1]. Despite this, the extracellular loop 2 (ECL2) disulphide bond pattern is uniquely conserved among all the GPCR subfamilies as it is required to maintain their 7TM fold [1]. Class B and other GPCRs demonstrate a very broad range of activities and respond to molecules of different sizes, from small molecules to peptides and proteins. Any changes in GPCR structures or in GPCR signalling can lead to various diseases, ranging from metabolic and neurological disorders to cancer [2]. Disruption in GPCR signalling causes derangement of upregulation and downregulation processes that leads to, e.g., carcinogenesis [3,4]. GPCR kinases (GRKs) terminate GPCR signalling by phosphorylation, which is known as desensitization. Phosphorylated GPCRs are bound to arrestins, regulating their trafficking by uncoupling them from their G proteins [2]. β-arrestins are reported to promote the internalization of GPCRs through endosomes following their uncoupling from G proteins [5,6,7].

Among class B GPCRs, VPAC1, VPAC2, and PAC1 receptors are known for their role in smooth muscle relaxation and regulation of exocrine and endocrine secretions. They are activated by two related neuropeptides [7,8], vasoactive intestinal peptide (VIP) and pituitary adenylate cyclase-activating peptide (PACAP) of common, helical conformations [9,10,11]. VPAC1 and VPAC2 show similar affinity towards VIP and PACAP whereas PAC1 demonstrates higher affinity towards PACAP and lower affinity towards VIP [8,12]. VIP and PACAP belong to a closely related group of peptides also including peptide histidine-isoleucine (PHI) and its human analog peptide histidine-methionine (PHM), secretin, and growth hormone-releasing hormone (GHRH). 

VIP was originally isolated in 1970 from pig small intestine as a 28-amino acid peptide able to induce vasodilatation and reduce arterial blood pressure [13]. Subsequent distribution studies revealed that VIP is widely distributed throughout the body, including in the central nervous system (CNS) and in peripheral nerves, in numerous tissues in the periphery as well as in the immune system [14]. Pituitary adenylate cyclase-activating polypetide (PACAP) owes its name to its discovery in 1989, from ovine hypothalamus extract, based on its ability to stimulate cAMP production in rat pituitary cells. This novel 38-amino acid peptide was therefore named PACAP-38 [15]. A few months later, a second peptide capable of stimulating adenylate cyclase activity was isolated from ovine hypothalamus extract. This 27-amino acid peptide was demonstrated to correspond to the N-terminal portion of PACAP-38 generated thanks to an internal cleavage-amidation site (Gly^28^-Lys^29^-Arg^30^) [15]. PACAP-27 and PACAP-38 were suggested to be related to VIP due to the high level of homology (68%). The first studies aiming to study PACAP distribution mainly focused on the CNS. Overall, PACAP-38 was found to be the major form. The highest amounts of PACAP are found in the hypothalamus, however, many other brain regions express significant concentrations of the peptides. As mentioned above, PACAP is also expressed in numerous peripheral tissues and in the immune system. As in the brain, PACAP-38 accounts for the major amount of total PACAP content [14,16].

VIP, PACAP, and their receptors are involved in growth, development, immune response, cardiac rhythm, neuronal as well as endocrine control, along with digestion, respiration, reproduction, and the cardiovascular system [17]. VIP and PACAP peptide hormones are secreted by intestinal, pancreatic, and hypothalamic tissues upon food intake, dysregulation of circadian rhythm or changes in social situations, especially affecting women (post-traumatic stress disorder). While intestinal secretion of VIP increases motility and lowers blood pressure due to a vasodilating effect, hypothalamic secretion of PACAP induces, e.g., migraines. In the last two decades, VIP and its receptors have emerged as potential pharmacological targets for various diseases, including asthma, brain stroke, chronic inflammation (Crohn’s disease, rheumatoid arthritis, septic shock, multiple sclerosis, etc.), neurodegenerative disorders, as well as cancers [17].

It was observed that increased expression of VPAC1 receptors is associated with colon, breast, lung, thyroid, and prostate cancers, whereas only a few cancers, like leiomyomas and gastrointestinal stromal tumors, showed overexpression of VPAC2 during cancer progression. Hence, VPAC1 and VPAC2 are considered not only as potential targets for chemotherapies but also for cancer diagnosis [12]. PAC1 is involved in diverse biological processes as its agonist PACAP acts as a neurotransmitter in regulating hormone secretion (adrenaline, insulin, growth hormone, follicle-stimulating hormone, luteinizing hormone, prolactin, and adrenocorticotropic hormone), tear secretion, vasodilation, and immunosuppression. It is also involved in neuroprotection in case of cerebral brain ischemia, Parkinson’s disease, spinal injury, etc. [18,19].

VIP and PACAP differ by their distribution in the CNS, with only a few cases of their co-localization reported [14,16]. In contrast, in many peripheral tissues and in the immune system, co-expression of VIP and PACAP is well documented. In general, the distribution of VIP and PACAP throughout the organism correlates with their pleiotropic effects, such as regulation of intestinal motility and secretion, exocrine and endocrine secretions, neuronal survival and plasticity, learning, control of circadian rhythms, and homeostasis of the immune system [14,16].

VPAC1 is expressed in the brain, T lymphocytes, liver, lungs, and intestines, as well as in tumors. VPAC2 receptors are mostly observed in the hippocampus, spinal cord, and smooth muscle tissues [20,21]. PAC1 receptors are predominantly found in the adrenal medulla and neuroendocrine neoplasms [22,23]. As mentioned above, VPAC2 is rarely expressed only in a few tumors, such as leiomyomas and gastrointestinal stromal tumors [24,25], in contrast to cancer-related, frequent overexpression of VPAC1. PAC1 is expressed in gliomas, neuroblastomas, and pituitary adenomas, as well as in endometrial cancers [24,25]. Considering the variation in expressions of VIP and PACAP receptors in both normal as well as cancer tissues during different malignant conditions, these receptors have gained a lot of attention also as a molecular signature for targeted imaging [12].

VIP, PACAP, and their receptors are involved in many overlapping processes in the gastrointestinal, immune, reproductive, respiratory, cardiovascular, and endocrine systems. VIP and PACAP improve gut motility, acting as vasodilating hormones, as well as exerting a hypophysiotropic effect on pituitary hormone secretion [26,27,28,29]. Apart from the role of VIP and PACAP receptors in physiological processes, they are also known to be involved in the progression of malignancies and tumor growth stimulation along with angiogenesis because of transactivation of epidermal growth factor receptor (EGFR) and the expression of vascular endothelial growth factor (VEGF) [30,31]. The increase in VIP/PACAP-mediated signalling due to overexpression of their receptors represents a standard hallmark in different cancers [12]. An increased level of VIP/PACAP and their receptors in cancer and variation of their levels observed during cancer progression makes it the best candidate not just for detection of different cancers but also for their diagnosis [32]. For example, the addition of VIP to lung cancer cells elevated cAMP levels, leading to the activation of transcription factors promoting expression of nuclear oncogenes and growth factors along with a rise in VEGF secretion and tumor angiogenesis through the cAMP/PKA and PI3K signalling pathways. As was mentioned above, VIP and PACAP receptors are used in molecular imaging at different cancer stages, including tumor formation and progression [30,31,33,34]. They are useful in the receptor-assisted imaging of tumors through non-invasive methodologies, including antibodies or their fragments, peptides, and their analogs, but also non-peptide small molecules to evaluate the effects of drug therapies in vivo [12,35].

Since the effects of VIP and PACAP are regulated by VIPRs, various analogs of them are used for the treatment therapies of physiological problems associated with gastrointestinal, immune, reproductive, respiratory, cardiovascular, and endocrine systems, as well as cancer malignancies. Somatostatin receptor-based agent (111)In-DTPA-pentetreotide (OctreoScan, Mallinckrodt Medical) is approved for clinical somatostatin receptor imaging, 90Y-labeled octreotide was reported to control the growth of medium-sized tumours in a transplantable rat tumour model [36] and 123I-labeled VIP is already reported to scan patients with different cancers, including gastrointestinal adenocarcinomas [37]. Different analogs of VIP, like VIPhyb (PAC1 antagonist) as well as (SN)VIPhyb (VPAC1 antagonist) and VIP-ALALA-E and VIP-LALA-E (VPAC1 agonists), are reported to inhibit various cancers [38], whereas Ro 25-1553 [39] and BAY 55-9837 (VPAC2 agonist) have been reported to be effective against bronchial asthma and insulin secretion, respectively [12]. In addition, VIP and PACAP-regulated activity-dependent neuroprotective protein (ADNP), which in turn regulates over 400 genes important for brain formation, has been mentioned in the treatment of cognitive impairment [12].

Targeting VIP and PACAP receptor signalling is considered as a new approach against multiple sclerosis [40]. Different agonists for the VPAC1 receptor, [K15, R16, L27]VIP(1-7)/GRF(8-27), [Ala11,22,28]VIP, [L22]VIP, [R16]PACAP(1-23), and LBT-3393, have been reported so far. Furthermore, antagonists for VPAC1, like PG97-269, have been approved for treatment. Agonists for VPAC2 include the abovementioned cyclic peptides Ro25-1553 and Ro25-1392 along with other peptides: BAY 55-9837, hexanoyl [A19,K27,28]VIP, recombinant RBAYL, and LBT-3627 have already been tested as drugs. For VPAC2, only two antagonists, PG99-465 and VIpep-3, are known at present [41]. To our knowledge, until now, 10 clinical trials have been completed involving VIP, out of which one was terminated, one was suspended, and one has an unknown status. The rest of the seven completed trials referred to pulmonary arterial hypertension, chronic obstructive pulmonary disease, migraine, and neurological disorders, etc. Similarly, 10 clinical trials involving PACAP-27/PACAP-38/VIP have been completed, out of which nine were associated with migraine while one involved nephrotic syndrome (https://clinicaltrials.gov/ct2/home (accessed on 3 December 2021)).

## 2. Molecular Switches in VIP and PACAP Receptors

Recent advances in cryo-EM brought insights into the active state conformations of VPAC1 and PAC1 receptors [42,43,44]. Previous studies described only conformations of extracellular domains of these receptors in solution [9] and in crystal [10], yet there were discrepancies between them regarding the peptide agonist binding site [45]. Inactive state conformations of VIP and PACAP receptors remain to be determined. Nevertheless, with current high-throughput methodologies for GPCR structure prediction [46,47] and easy validation of homology models based on the ROC–AUC assessment of virtual screening results [48,49], models of VPAC1, VPAC2, and PAC1 of a quality sufficient for drug discovery purposes have been obtained [50]. Such a high level of model precision enabled investigation of the mechanism of receptor activation through analysis of differences between inactive and active state conformations (see below).

**Figure 1:**
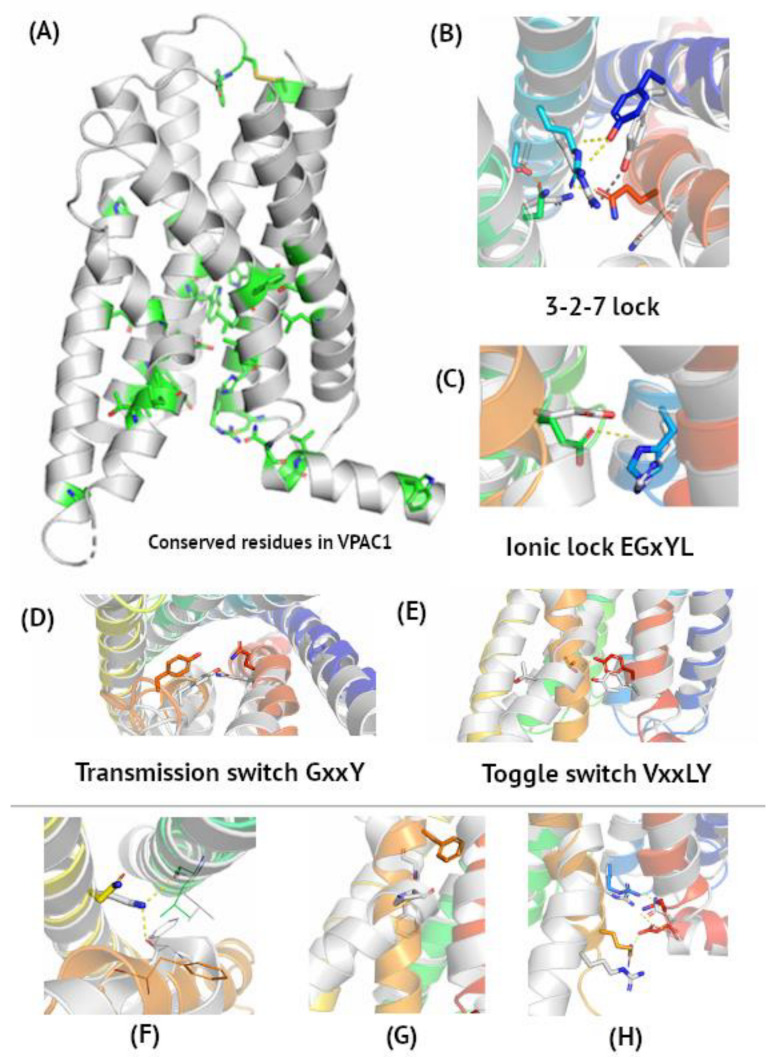
*Microswitches in VIP and PACAP receptors.* Panel (**A**), with the VPAC1 active conformation (6VN7), represents the most conserved amino acids, according to multiple sequence alignment of class B sequences in Supplementary Figure S2. Panels (**B**–**E**) represent molecular switches involved in VIP and PACAP receptor activation corresponding to class A microswitches notation (toggle switch, ionic lock, etc.) [54]. Here, a superposition of the VPAC1 active cryo-EM structure (grey, PDB id: 6VN7) and inactive VPAC1 homology model based on GLP-1R (PDB id: 5VEW, blue-to-red) was shown. Panels (**F**–**H**) represent other non-covalent interactions formed or broken during receptor activation. (**B**) 3-2-7 ionic lock joining TM3, TM2, and TM7 in the inactive receptor conformation. Interactions Asn3.43–Ser2.56 and Arg2.60–Gln7.49 are broken upon activation, while Arg2.60–Tyr1.47 is maintained. (**C**) His2.50–Glu3.50 ionic lock brakes upon activation. (**D**) Tyr6.53 couples with Gln7.49 upon activation. (**E**) Thr6.42–Tyr7.57–Tyrosine toggle switch turning into ‘off’ position upon activation. (**F**) Upon activation, Asn5.50 forms hydrogen bonds with the main chain atoms of TM3 and TM6. (**G**) A global toggle switch involving bending and moving of TM6 facilitated by Gly and Pro residues in the TM6 kink. (**H**) An intracellular hydrogen bonding network joining TM2, TM6, TM7, and H8 breaks upon activation due to movement of Arg6.40 and Glu8.49.

Most conserved residues in VPAC and PAC1 receptors are in TM6 and TM7, and in their central and bottom (intracellular) part (see Figure 1). These regions are crucial for receptor activation and change their conformations between ‘on’ and ‘off’ states while binding G protein subunits. TM1, TM4, and the upper (extracellular) part of VIP and PACAP receptors demonstrate higher sequence variation, except for Cys in a conserved, ECL2 disulphide bond. This sequence variation in the extracellular part of the receptor contributes to its relatively weak agonist selectivity. VPAC receptors bind both VIP and PACAP [45], but also other class B peptide agonists (e.g. secretin) due to a common evolutionary link between them (see Supplementary Figure S1). These diverse chemical stimuli trigger the same response in the conserved intracellular region of the receptor, leading to, e.g., a rise in cAMP levels, PKA activation, increasing Ca2+ extrusion, induction of hyperpolarization, and, finally, arterial vasodilation [51]. The same mode of signal transduction observed for VIP, PACAP, and their receptors and other class B members justifies this sequence conservation in the intracellular G protein or arrestin binding regions. On the other hand, relatively weak selectivity towards endogenous agonists justifies the sequence variability in extracellular regions of these receptors.

According to de Graaf et al. [52], the most conserved residues in each TM helix of class B GPCRs are as follows: TM1–Ser1.50, TM2–His2.50, TM3–Glu3.50 in the EGxYL motif (corresponding to R in ‘E/DRY’ of class A GPCRs), TM5–Asn5.50, TM6–G6.50, TM7–G7.50 in the SFQG motif. Based on multiple sequence alignment of class B sequences (Supplementary Figure S2), TM4–W4.50 in the GWGxP sequence motif can also be added [53], similarly to W4.50 in class A GPCRs. The GWGxP motif stabilizes TM2, TM3, and TM4 interactions like in CRF1, another class B GPCR [53], yet no significant changes were observed in this region when comparing active and inactive state conformations. Additionally, Leu2.53, Phe2.57, Asn3.43, Trp3.46, P4.53, P5.42, Thr6.42, Leu6.43, Leu6.45, Phe7.48, and V7.53 are conserved among class B members (Supplementary Figure S2). Here, Ballesteros–Weinstein numbering was used for naming residues. These conserved residues are involved in receptor activation (see Figure 1) and change their conformations upon agonist binding. 

Receptors from class A (rhodopsin-like GPCRs) undergo structural rearrangements leading to receptor activation through a series of local conformational changes, so-called molecular switches [54]. In principle, upon binding, agonists interact with residues in these molecular switches and thus initiate activation and signalling cascades. Recently, it has been shown that receptors from other classes—F [55], C [56] and also B [52,53,57]—also undergo a similar pathway of activation, from local to global structural rearrangements. These global structural rearrangements involving TM helix deformations required for G protein binding are called ‘macroswitches’ [58] in contrast to local rearrangements (‘microswitches’) involving only changes of side chain rotamers upon agonist binding and receptor activation. 

There are four main microswitches in class A GPCRs: 3-7 lock, ‘E/DRY’ ionic lock, ‘CWxP’ transmission switch, and ‘NPxxY’ Tyrosine toggle switch [54]. In class B GPCRs, slightly different amino acids are involved in the activation initiation, yet the mechanism of the activation initiation is like that of class A GPCRs [52,53]; namely, the transmission switch involves the movement of Tyr6.53 towards Gln7.49 upon activation (Figure 1B). Instead of Arg in the ‘E/DRY’ motif of class A GPCRs, Glu3.50 interacts with His2.50 in the inactive receptor conformation (Figure 1C) [53]. Interactions between Thr6.42 and Tyr7.57 break upon activation (Figure 1D), like in a tyrosine toggle switch of class A GPCRs. This tyrosine toggle switch was also described in PAC1 by Wang et al. [44]. The 3-7 ionic lock observed in rhodopsin changes into the 3-2-7 ionic lock in VIP and PACAP receptors (Figure 1E). Thus, inactive state interactions between TM3 and TM7 are mediated in class B GPCRs through TM2 (Asn3.43–Ser2.56 and Arg2.60–Gln7.49). Upon PACAP binding by VPAC1, these interactions break to enable its new location close to Phe3.44 [42]. The position of Arg2.60 in this ionic lock is stabilized by interactions with Tyr1.47, observed in both active and inactive states. Interestingly, PACAP-38 binds to both of these residues, Tyr1.47 and Arg2.60, in PAC1, but it still confirms a similar mechanism of activation of all three VIP and PACAP receptors [44]. Mutations of Arg2.60 impaired ligand-induced activation [42], confirming its involvement in the 3-2-7 ionic lock. On the other hand, breaking of the Arg2.60–Gln7.49 lock is needed for Gln7.49 to stabilize the kink conformation of TM6 in active VPAC1 through interactions with Tyr6.53 [42].

The TM6 global toggle switch is also observed in class B GPCRs. Its change is facilitated by conserved Gly6.50 and Pro6.47 (Figure 1F), well-known helix breakers [59]. During this movement, the most distant C atom (Cζ) in Phe6.49 changes its position by as much as 12 Å. Except for the microswitches described above, VIP and PACAP receptor activation involves the following changes in non-covalent interactions. Asn5.50 forms hydrogen bonds with the main chains of TM3 and TM6, which stabilizes the active conformation (Figure 1G), also mentioned in [42]. An intracellular hydrogen bond network involving TM2, TM6, TM7, and H8 breaks upon activation to enable interactions with G-alpha subunits (Figure 1H) and the Arg6.40–Glu8.49 interaction is lost. Mentioned above residues are involved in three layers of polar networks which reorganize upon activation and G protein binding [44].

## 3. Signalling Pathways Activated by VIP and PACAP Receptors

**Figure 2:**
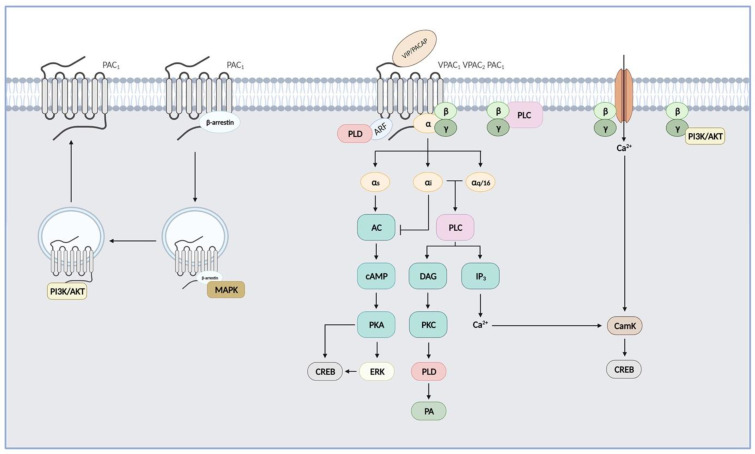
*VIP and PACAP receptor-mediated signalling pathways*. PAC1 and VPAC receptors are preferentially coupled to Gαs, leading to activation of adenylate cyclase (AC), subsequent cAMP production, and activation of protein kinase A (PKA). PKA may in turn activate extracellular signal-regulated kinases (ERKs). These three class B receptors can also activate the phospholipase C (PLC) pathway after coupling to Gαi, Gαq, or Gα16 and stimulate calcium levels and protein kinase C (PKC). Released Gβγ can activate PLC and phosphoinositide 3-kinase (PI3K/Akt) pathways, and Ca^2+^ entry into the cell through receptor-operated Ca^2+^ ion channels. PAC1 and VPAC receptors may also activate phospholipase D (PLD) through the ADP-ribosylation factor- (ARF) or a PKC-dependent mechanism. cAMP response element-binding protein (CREB) signalling was also demonstrated following activation by kinases acting downstream of PAC1 and VPAC receptors, such as PKA, CamK (Ca²⁺/calmodulin-dependent protein kinase), and ERK. Internalized receptors may also activate G protein-independent signalling pathways, as seen for PAC1 and activation of MAPK through a β-arrestins-dependent mechanism and for PI3K/Akt.

Like all GPCRs, upon agonist binding, VIP and PACAP receptors undergo conformational changes that allow interactions of their cytoplasmic-facing regions with heterotrimeric G proteins promoting exchange of GDP for GTP on the Gα subunit. This initiates the dissociation of the GTP-bound Gα subunit from the Gβγ dimer and activation of downstream effector pathways. Signals are terminated following hydrolysis of Gα-bound GTP and reformation of the inactive trimeric G protein complex [60]. VIP and PACAP receptor-mediated signalling pathways were shown in detail in Figure 2.

### 3.1. Adenylate Cyclase/cAMP Pathway

VIP and PACAP receptors are preferentially coupled to Gαs, leading to activation of adenylate cyclase and subsequent cAMP production that accounts for most of their physiological effects, such as smooth muscle relaxation, stimulation of endocrine and electrolyte secretions, etc. Accumulation of intracellular cAMP also leads to activation of protein kinase A (PKA), which contributes to the neurotrophic functions of VIP and PACAP, such as the stimulation of neuronal stem cells and neuroblast differentiation, as well as modulation of synaptic plasticity following the activation of channels by PKA [16,61]. PKA may also activate the ERK signalling pathway to promote cell proliferation, as seen for instance in pituitary, prostate cancer, and colorectal cancer cell lines [62,63,64]. PACAP also simulates the proliferation of glial cells through a cAMP/ERK-dependent but a PKA-independent mechanism [65]. The cAMP/PKA/ERK pathway may also lead to inhibition of caspase 3 and increase of Bcl-2 expression, thus promoting cell survival [16,61,66]. PKA activation is also responsible for most of the anti-inflammatory properties of VIP and PACAP by regulating several signalling pathways and transcription factors, thus increasing anti-inflammatory cytokines and reducing pro-inflammatory cytokine production [67].

### 3.2. PLC/Calcium Pathway

Several studies also reported that PAC1 and VPAC receptors were able to activate the phospholipase C (PLC) pathway and increase intracellular calcium levels ([Ca^2+^]_i_), either in cells endogenously expressing these GPCR receptors or in transfected cell lines. It has been shown that PAC1-mediated activation of PLC involves exclusively the Gq-mediated mechanism [68]. While for VPAC1 and VPAC2, [Ca^2+^]_i_ increase relies on both Gαi and Gαq coupling [68,69]. Moreover, pertussis toxin (PTx)-sensitive, thus Gi-mediated, mechanisms leading to PLC activation seem to be different for VPAC1 and VPAC2. Indeed, interactions between VPAC1 and Gαi were demonstrated and it was confirmed that the VPAC1-mediated [Ca^2+^]_i_ increase is not affected by the chelation of extracellular calcium [69], whilst the PTx-sensitive activation of PLC by VPAC2 relies on a Gβγ-dependent mechanism and on Ca^2+^ entry through receptor-operated Ca^2+^ channels [68]. Finally, it was also observed that both VPAC1 and VPAC2 can couple to Gα16, a G protein of the Gαq family, which enables the coupling of a wide variety of receptors to PLC and whose expression is restricted to hematopoietic cells (except for the mature B cells) [70]. PAC1 and VPAC receptor-dependent activation of PLC/calcium pathways was shown to be responsible for some of their biological activity, such as cell migration, secretion of neurotransmitters and neurohormones (calcium influx and CamK-dependent mechanisms), and stimulation of glial cell differentiation (via a PKC-dependent mechanism). In some studies, the peptide doses to obtain a PLC activation, or other alternative signalling pathways, were relatively high as compared to receptor affinity. Therefore, the physiological relevance of these alternative signalling pathways activated by VIP and/or PACAP remains conjectural. However, in various pathologies, including cancers where an overexpression of these receptors was identified, the activation of these alternative signalling pathways could be responsible for the malfunction of cells [16,61].

### 3.3. CREB Pathway

CREB (cAMP response element-binding protein) is a transcription factor activated by several kinases, including kinases acting downstream of the activation of PAC1 and VPAC receptors, such as PKA, CamK, and ERK. It is therefore not surprising that some studies demonstrated VIP- and PACAP-mediated activation of CREB in cells endogenously expressing these receptors or in transfected cell lines. The activation of CREB by PACAP and VIP is involved in their capability to increase cell differentiation and secretion of neurotransmitters and neurohormones in the hypothalamus [14,16,61]. 

### 3.4. G Protein-Independent Pathways

Additional coupling events that are not G protein-mediated may also elicit auxiliary signals. For instance, PAC1 and VPAC receptors can also activate phospholipase D (PLD). PLD activation by the PAC1 hop1 splice variant and VPAC receptors is sensitive to brefeldin A, an inhibitor of ADP-ribosylation factor (ARF) known to act as a direct activator of PLD [71]. Biological effects linked to the PLD pathway have not been investigated so far. In pancreatic β cells, VIP-induced sustained stimulation of insulin secretion is mediated by the activation of the phosphatidyl inositol 3 kinase (PI3K) by VPAC2 receptors [72]. Similarly, neuroprotective effects of PACAP in the adult brain and its capability to stimulate dendrite outgrowth relies on PI3K activation [16]. Finally, VIP-induced neuroendocrine cell differentiation of LNCaP cells also involves a PI3K-dependent mechanism [63].

Besides coupling to the effectors, GPCR activation by agonists also initiates the process of receptor desensitization, an adaptive response contributing to rapid fade of G protein signalling. This process starts with phosphorylation of the receptor amino acids located in intracellular loops or C-terminus by activity-dependent kinases (PKA and PKC) and/or by GPCR kinases (GRKs). GRK-mediated phosphorylation of the receptor promotes the high-affinity binding of β-arrestins to the receptor, which both sterically interdicts further coupling of G proteins and may act as a signal transducer to activate, for instance, MAPKs, PI3K, and Akt. β-arrestins are also able to bind proteins of the endocytic machinery, including clathrin and adaptor protein AP2, and promote receptor internalization [60].

Like most GPCRs, VIP and PACAP receptors are rapidly phosphorylated and internalized upon agonist exposure. Studies demonstrated that the main kinases involved in agonist-induced receptor phosphorylation were PKC for PAC1 receptors [73] and GRK (and PKA to a lesser extent) for VPAC receptors [74,75,76]. As mentioned before, internalized receptors may also activate G protein-independent signalling pathways, as seen for PAC1 and activation of the PI3K/Akt and MAPK pathways [77,78]. To date, such a mechanism has not been described for VPAC receptors. It was suggested that specific receptor conformations required for either activation, phosphorylation, or internalization could be different, so this difference between PAC1 and VPAC receptors is highly likely [79]. These findings indicate that beside the specific expression and distribution of VIP, PACAP, and their receptors, the fine tuning of their biological activity, and therefore the way to modulate it, also rely on the diversity of signalling pathways they can trigger.

### 3.5. VIP and PACAP Potency to Stimulate Signalling Pathways

In addition to the development of the receptor-selective ligands, another approach to developing new GPCR therapeutics has been to identify ligands favouring specific conformations of the receptor that activate only some signalling pathways (so-called biased signalling) [60]. One example is the discovery of μ-opioid receptor (MOR) agonists that preferentially induce G protein signalling over the β-arrestin recruitment. Thus, side effects of opioid analgesics can be reduced [80]. Such functionally selective ligands are called biased ligands.

**Figure 3:**
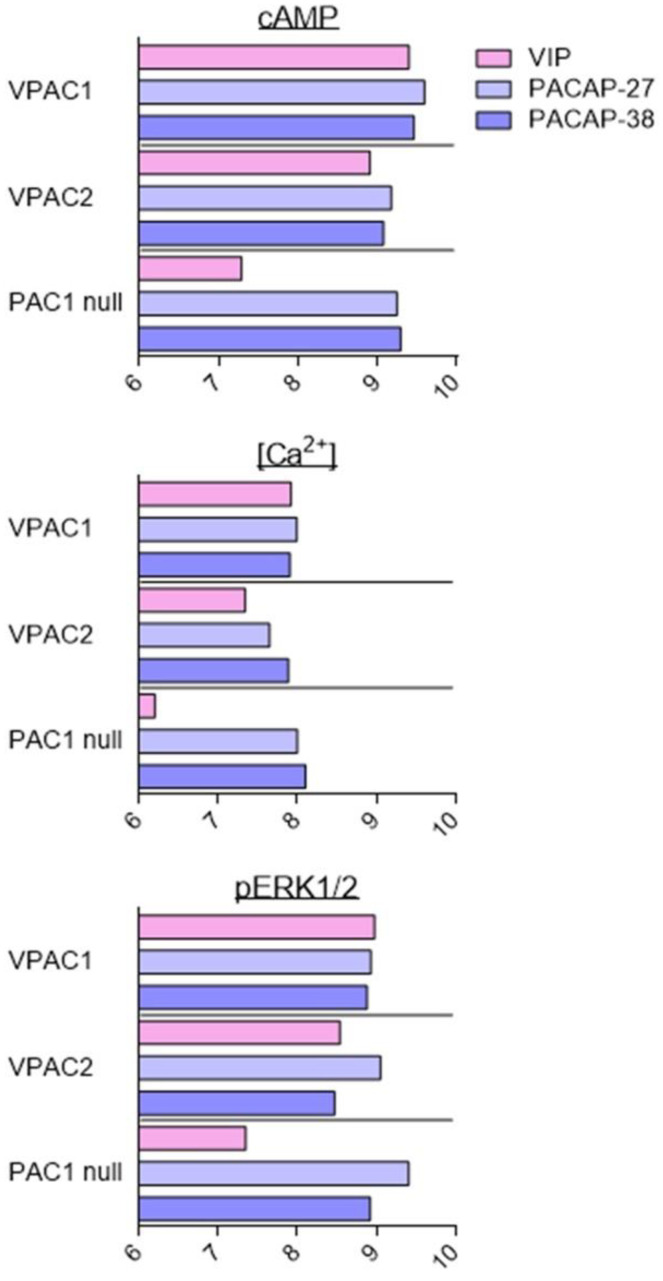

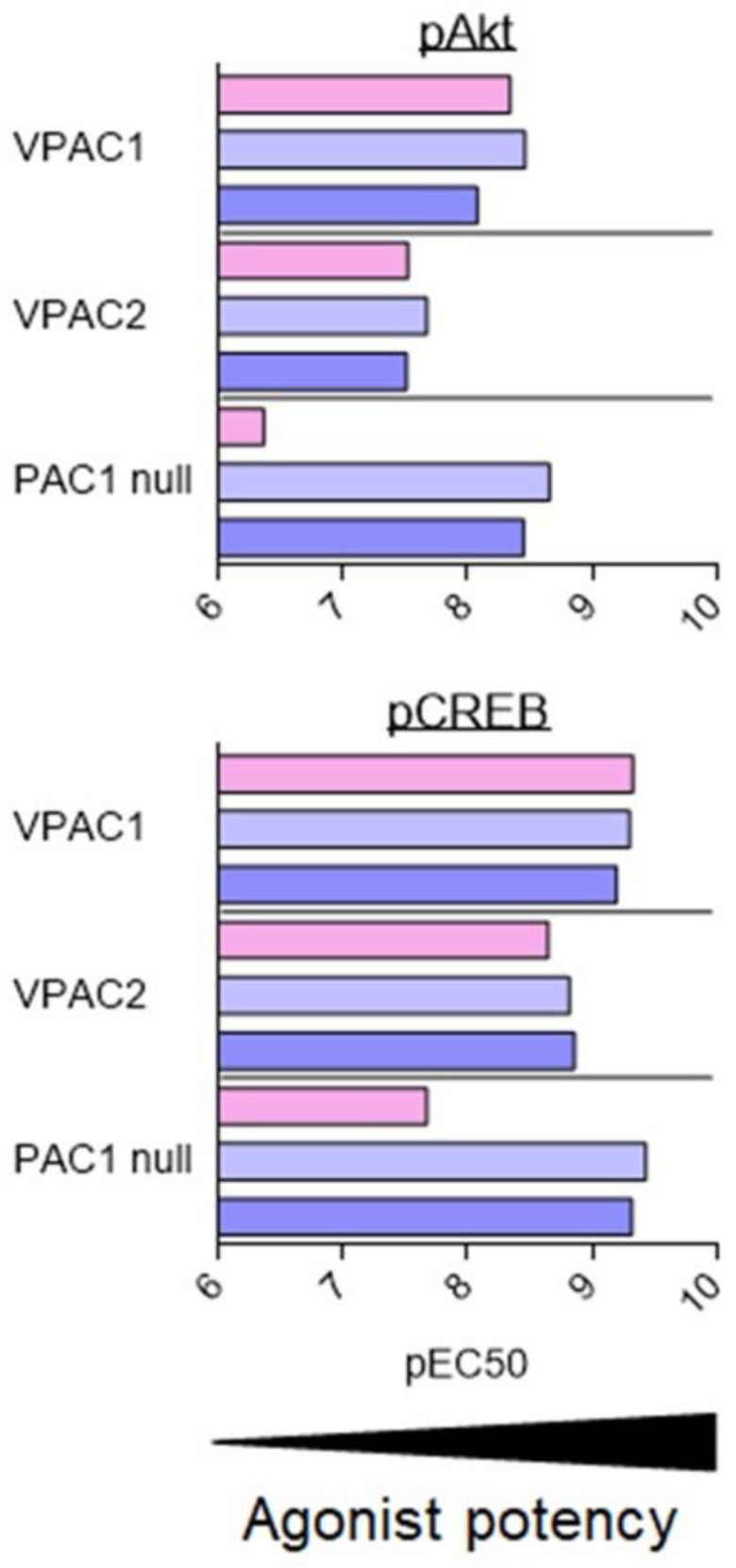
*Stimulation of signalling pathways in stably transfected CHO cell lines.* Summary of VIP (pink), PACAP-27 (light blue), and PACAP-38 (dark blue) potency, evaluated by pEC50 values (−logEC50 (M)) for stimulation of signalling pathways in cells stably transfected with human VPAC1, VPAC2, or PAC1 null receptors. Data collected from [81,82].

As described above, VIP and PACAP receptors can activate a broad range of signalling pathways. Overall, the fine analysis of the data suggests that agonists are more potent for inducing production of cAMP than other second messengers. However, because of the diversity of cell types, receptor expression levels, receptor types, and assays used in the different studies, it is difficult to conclude that the observed differences are significant and determine what they imply. To date, only two studies have tried to address this question with a systematic comparison of PAC1 and VPAC receptor signalling using stably transfected cell lines [81,82]. These studies evaluated the effect of VIP, PACAP-27, and PACAP-38 on CHO cells stably transfected with human VPAC1, VPAC2, and PAC1 null (often considered as the “reference” PAC1 receptor). Five signalling pathways were investigated: cAMP, PLC/Ca^2+^, ERK, Akt, and CREB (Figure 3). In agreement with the low-affinity binding of VIP to the PAC1 null receptor, VIP was always less potent than PACAP-27 and PACAP-38 to induce PAC1 signalling. Whilst overall, VIP and PACAP were equipotent to activate VPAC receptors. VIP was only slightly less potent than PACAP-38 to stimulate the VPAC2-mediated PLC/Ca^2+^ pathway. Subtle differences were also observed between PACAP-27 and PACAP-38. PACAP-27 was a little bit more potent than PACAP-38 to stimulate VPAC1-mediated Akt phosphorylation or PAC1-mediated ERK phosphorylation. Interestingly, for these three receptors, VIP and PACAP were 10 to 100 times less potent to activate the Akt and PLC/Ca^2+^ pathways than cAMP production or ERK and CREB phosphorylation [81,82]. 

Based on the above data, it’s likely that VIP and PACAP may act as natural biased ligands for their own receptors and that blocking of a specific signalling pathway may be influenced by the level of endogenous ligand present. However, recombinant expression models, due to the overexpression of the receptors, may not exactly recapitulate what is indeed happening in physiological systems. One-hundred-fold differences in agonist potencies suggest that this aspect must be also considered, especially for in vivo experiments.

## 4. Impact of PAC1 and VPAC Receptors Genetic Diversity on Signal Transduction 

### 4.1. The VIP and PACAP Genes

The human VIP gene (VIP) was cloned in 1985 and is composed of seven exons [83]. Its cDNA encodes for a 170-amino acid precursor named prepro-VIP. Like several other neuroendocrine peptides, processing of prepro-VIP yields the 28-amino acid peptide VIP encoded by exon 4 and an additional bioactive peptide encoded by exon 3, the 27-amino acid peptide histidine methionine (PHM) in humans, or the peptide histidine isoleucine (PHI) in rodents [84]. PHM shares 48% amino acid homology with VIP.

The human PACAP gene (ADCYAP1) was cloned in 1992 [85]. The organisation of this gene is much like that of the VIP gene. It is composed of five exons and encodes for a peptide precursor of 176 amino acids, prepro-PACAP. This precursor is cleaved and modified to produce PACAP-27 and PACAP-28 (encoded by exon 5), as well as a third peptide of 29 amino acids (PRP for PACAP-related peptide encoded by exon 4) that shares only 20% homology with PACAP [85]. PRP shares 40% homology with PHI. Both VIP and PACAP promoters possess several cassettes that regulate gene expression. These include cAMP response element (CRE), cytokine-responsive region, phorbol ester element, hormone response element, etc. PACAP itself can also enhance the transcription of VIP and PACAP genes [83,85].

### 4.2. Sequence Conservation of VIP, PACAP, and Their Receptors

**Figure 4:**
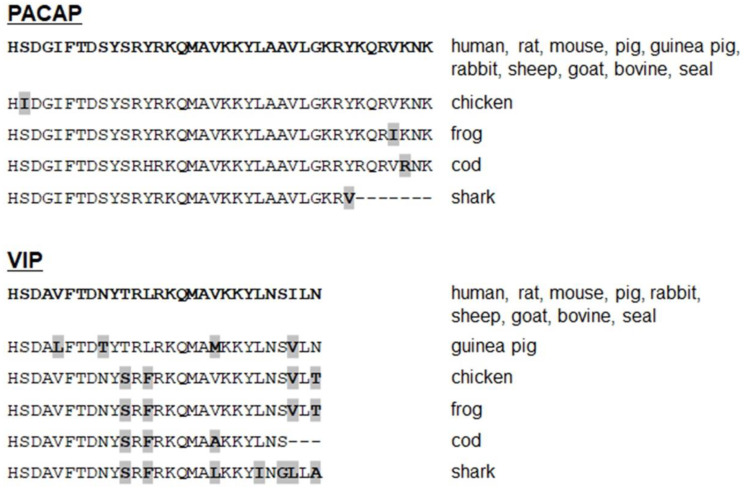
*Comparison of PACAP and VIP amino acid sequences from various vertebrate species.* Amino acids that differ from human sequences are in bold and highlighted, and those which are missing are indicated with dashes.

VIP and PACAP are the most conserved peptides among the secretin–glucagon peptide family. PACAP-27 is fully conserved in all vertebrate classes, except for the chicken sequence with only one amino acid substitution compared to the human sequence. The C-terminal 28–38 region of PACAP, which is less important for its biological activity, is more variable. However, differences are small as they correspond to only a few amino acid substitutions and were only found in some non-mammalian vertebrates (Figure 4). PACAP-27 was also found in tunicates that were supposed to be ancestral for vertebrates, with only one different amino acid from human PACAP [16]. The VIP sequence is identical in all mammalian species except for the guinea pig whose VIP sequence differs by four amino acid substitutions. A few substitutions are also found in other vertebrates, such as chicken, fish, and amphibia. Of interest, these substitutions do not affect the five-amino acid-long N-terminal region of VIP, which is known to be crucial for receptor activation in all species except for the guinea pig (Figure 4).

**Figure 5:**
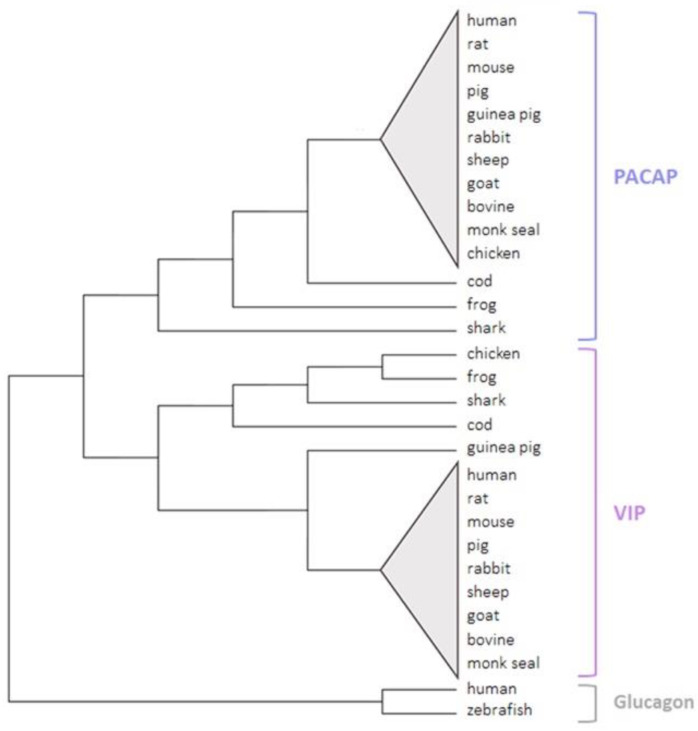
A *phylogenetic tree of PACAP and VIP in the vertebrate sub-phylum.* Sequences were collected from the UniProt and the NCBI databases. The tree was generated via MEGA11 software using the maximum likelihood and bootstrap methods. Zebrafish and human glucagon peptides were used as out-groups.

The unique conservation of the peptide sequences in vertebrates (Figure 5), particularly the PACAP sequence, suggests that PACAP and VIP are involved in major biological functions in all vertebrate species. Moreover, this finding that PACAP has been so well conserved over hundreds of million years of evolution also supports the hypothesis that the PACAP gene may constitute the ancestral gene of the VIP/PACAP family of peptides [16,86].

### 4.3. Gene Co-Occurrence Analysis

**Figure 6:**
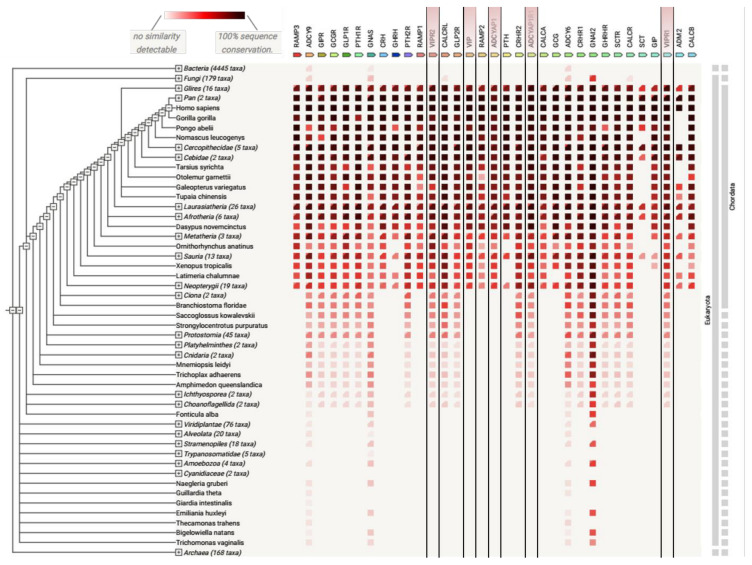
*Analysis of protein co-occurrence in various species.* Co-occurrence of class B GPCRs and proteins interacting with them (RAMP1-3, GNAS, etc.) obtained with the Search Tool for the Retrieval of Interacting Genes/Proteins (date of accession: 3rd December 2021) [87]. VIP, PACAP, and their receptors were marked with lines. Among Bacteria, Fungi, Eukaryota, and Archaea species, class B members were predominantly distributed in more complex organisms (Eukaryota). This demonstrates a common evolutionary link among species.

The role of VIP and PACAP and their receptors in other organisms remains to be determined in detail, although the ancestral origin of these sequences in Agnatha have been already described by Ng et al. [86]. Nevertheless, an analysis of gene co-occurrence with the Search Tool for the Retrieval of Interacting Genes/Proteins [87] using the class B sequences currently available in databases showed the predominant distribution of VPAC2 in some eukaryotic species comparing VPAC1 and PAC1 but also other class B GPCRs (see Figure 6). For example, VPAC2 sequences in three species, Metatheria, Sauria, and *Ornithorhynchus anatinus*, were slightly more conserved with respect to the human VPAC2 sequence than VPAC1. In addition to VPAC2, GLP1R, PTH1R, PTH2R, CALCRL, and CRHR1 were more conserved in these species comparing other class B GPCRs (see Figure 6 and Supplementary Figure S3). According to data currently available [87] and settings adjusted rather to receptors than to their peptide agonists only, ancestors of VPAC1 and VPAC2 are visible in Ciona (belonging to Tunicata or previously: Urochordata) (see Supplementary Figure S3). However, Ng et al. [86] mentioned that some urochordate species included ancestral PRP-like and PACAP peptides and ancestral receptors for them. The above-described gene co-occurrence reflects the sequence evolution of VIP, PACAP, and their receptors. It also enables selection of the most suitable model organism for studying VIP and PACAP signalling pathways in vivo. For example, VPAC2 is less conserved in *Galeopterus variegatus* comparing VPAC1 and PAC1 and better conserved in *Ornithorhynchus anatinus* (see Figure 6 and Supplementary Figure S3). Yet these differences in sequence conservation among species can be also partly due to underrepresentation of data for some receptors and species deposited in [87].

## 5. VIP and PACAP Receptor Variants

Another level of complexity in describing the function of VIP and PACAP receptors comes from the different processing of genes coding for PAC1 (ADCYAP1R1) and VPAC (VIPR1 and VIPR2) receptors, giving rise to the subsequent production of splice variants [88]. To date, numerous PAC1, VPAC1, and VPAC2 splice variants have been identified. As properties of these splice variants may vary significantly among species, we will mainly focus on the human receptors. A detailed description of all known variants is available elsewhere [14,16,61,88].

The most important splice variants are listed in Supplementary Table S1, yet more sequences (more than 10 entries for both VPAC receptors) are already included in NCBI (see Supplementary Figures S4 and S5). VPAC1 and VPAC2 isoforms deposited in NCBI mostly differ in their N-terminal regions (see Supplementary Figures S4 and S5). Among sequences included in Uniprot, the most diverse are two isoforms of VPAC2 (below 80% sequence identity), while five PAC1 isoforms are identical but vary in length. Multiple sequence alignments of these splice variants are included in Supplementary Figures S6–S8.

### 5.1. VPAC1 Splice Variants

A five-transmembrane (5TM) form of human VPAC1 lacking 88 amino acids has been identified in human peripheral blood mononuclear cells. This alternatively spliced mRNA variant results from the skipping of exons 10 and 11, spanning over the third intracellular loop (ICL3), the third extracellular loop (ECL3), TM6, and TM7 [89]. This VPAC1 isoform was also detected in two malignant cell lines, Hut78 and Colo205 [89], and in human cDNA libraries [90]. Although expressed at the cell surface, this VPAC1-5TM variant fails to activate Gαs properly [89]. Two more short VPAC1 variants with alternative N-terminal domains have been identified in human cDNA libraries but have not been yet characterized [90].

### 5.2. VPAC2 Splice Variants

A five-transmembrane (5TM) form of human VPAC2 lacking 74 amino acids has also been identified in the human SUPT1 cell line. The pharmacological profile of this variant has not yet been examined [89]. Another VPAC2 variant resulting from the loss of exon 11 was identified in human lymphocytes. This variant binds VIP normally but displays reduced VIP-induced signalling [91]. Two more short VPAC2 variants with alternative N-terminal domains have been identified in human cDNA libraries but have not been yet characterized [90].

### 5.3. PAC1 Splice Variants

PAC1 null is often considered as the ‘reference’ PAC1 receptor and is encoded by exons 3 to 13 and 16 to 18 of the PAC1 gene (ADCYAP1R1). First splice variants of PAC1 have been isolated from rat brain cDNA and include insertions of one or two cassettes of 28 amino acids encoded by exon 14 (‘PAC1 hip’) or by exon 15 (‘PAC1 hop’) into ICL3. The splice variant bearing simultaneously the two cassettes is called ‘PAC1 hiphop’ [92]. These PAC1 variants have also been identified in other species, including humans, mice, and zebrafish [93,94]. Their expression is not restricted to SN (substantia nigra) as they also have been identified in the lungs, heart, jejunum, and adrenal gland [94].

Another set of PAC1 variants arise from the alternative splicing of the exons coding for the extracellular N-terminus. Three of them include a loss of 5, 21, or 57 amino acids corresponding to deletion of exons 5, 5 and 6, or 4 to 6, respectively. They were found in humans, rats, and mice, but they have been mainly detected in the CNS to date. Only in the adrenal gland and in the heart, small amounts of the PAC1 variant lacking exons 5 and 6 are expressed [92,95,96]. A fourth splice variant includes an insertion of 24 amino acids in the N-terminus of PAC1 that corresponds to the inclusion of exon 3a. This variant has been identified only in rats. Other variants with alterations in both ICL3 and N-terminus have also been identified [96,97]. Another splice variant, named PAC1 TM4, has been identified only in rats and differs from the PAC1 null sequence by small changes in TM2 and TM4. mRNA sequences encoding PAC1 TM4 were detected in rat CNS, pancreatic β-cells, and lungs [98].

**Figure 7:**
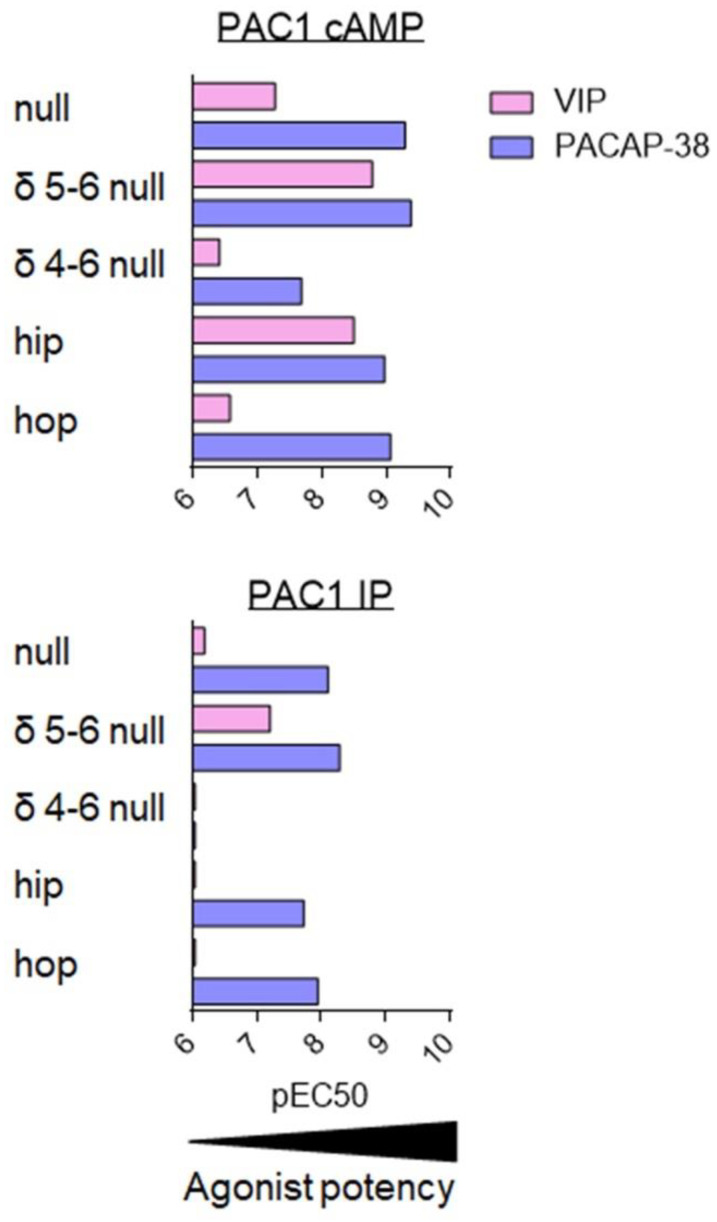
*Agonist potency for PAC1 splice variants.* Summary of VIP (pink) and PACAP-38 (dark blue) potency, evaluated by pEC50 values (-logEC50 (M)) for stimulation of AC/cAMP and PLC/IP pathways on cells stably transfected with human PAC1 null or selected splice variants. Data collected from [81,82,92,93,95,96].

All the PAC1 splice variants exhibit altered ligand selectivity and/or potencies as well as modified signalling properties. However, it is difficult to address the issue clearly as their phenotypes may differ between species and the studies performed so far have not always used the same endpoints. Therefore, we decided to focus on the human receptor only and variants for which at least two sets of independent data are available for the same ligand and the same signalling pathway. Figure 7 summarizes the data for human variants satisfying these criteria: PAC1 null, PAC1 δ5-6 null, PAC1 δ4-6 null, PAC1 hip, and PAC1 hop [81,82,92,93,95,96]. 

As compared to PAC1 null, the PAC1 δ4-6 null variant demonstrated one of the most altered phenotypes; namely, PACAP-38 and VIP potencies to increase cAMP were reduced by at least 10-fold and both ligands failed to stimulate inositol phosphate production (Figure 7). Poor activity of PACAP-38 and VIP agree with their low affinity for this variant [95]. The most striking difference was seen for the PAC1 δ5-6 null and PAC1 hip variants. Indeed, PAC1 is usually considered as the ‘PACAP-preferring’ receptor which binds VIP with low affinity. However, for these PAC1 variants, the VIP potency to increase cAMP concentration falls in the nanomolar range, suggesting that for PAC1 δ5-6 null and PAC1 hip variants both VIP and PACAP-38 are equipotent (Figure 7). This agrees with the higher affinity of VIP for these two PAC1 variants, which was evaluated by binding studies. These results may be physiologically relevant as they were obtained in both transfected CHO cells and in a cell line endogenously expressing PAC1 δ5-6 null [96]. Although still less potent than PACAP-38, VIP potency to stimulate inositol phosphate production was also increased in the case of PAC1 δ5-6 null, whilst VIP-mediated IP production in PAC1 hip was weak (Figure 7).

Despite their impact on agonist potency, splice variants of VIP and PACAP receptors do not vary in TM regions. Differences in sequences of PAC1 splice variants are in N-termini, ca. 50 residues, before the beginning of TM1, and, in ICL3, between TM5 and TM6, which is frequently long and disordered and substituted by lysozymes in crystal structures of GPCRs. ICL3 in HOP variants of PAC1 is longer (ca. 40 amino acids) than in VPAC1 null (ca. 10 residues) which most probably alters interactions between the receptor and the G-alpha subunit. VPAC1 and VPAC2 variants vary also in N-termini, yet much closer to the beginning of TM1 (ca. three residues in VPAC2).

## 6. Conclusions

Underrepresentation of active ligands and drugs modifying VIP and PACAP receptor signalling proves their complex nature. Relatively weak endogenous agonist selectivity and the impact of alternative splicing on observed agonist potency still prevent the efficient use of VPAC and PAC1 receptors as molecular targets in endocrine, metabolic, anti-inflammatory, and anti-cancer therapies [53]. Only recently [50], the first small-molecule negative allosteric modulator of these receptors has been discovered in a drug repositioning screen, opening a new therapeutic avenue. Recently released cryo-EM structures of VPAC1 and PAC1 in their active state have just provided a basis to study ligand recognition and receptor activation, leading to more efficient drug discovery in silico. Confirmed, but still not explained in all details, is the association of VIP/PACAP receptor signalling pathways with the regulation of excessive inflammatory responses, which presents a new approach for the treatment of chronic autoimmune diseases and acute inflammation states.

## Supplementary Materials

The following supporting information can be downloaded at: https://www.mdpi.com/article/10.3390/biomedicines10020406/s1, Figure S1: Network of all class B receptors and their peptide endogenous agonists, Figure S2: Sequence variation in TM helices of class B GPCRs, Figure S3: Co-occurrence of class B GPCRs and their peptide agonists in various species obtained from Szklarczyk et al. 2021, Figure S4: Isoforms of VPAC1 deposited in NCBI, Figure S5: Isoforms of VPAC2 deposited in NCBI, Figure S6: Multiple sequence alignment (MSA) of five VPAC1 sequences deposited in Uniprot, Figure S7: MSA of two VPAC2 sequences deposited in Uniprot, Figure S8: MSA of five PAC1 sequences deposited in Uniprot, Table S1: Major isoforms of VIP and PACAP receptors according to Uniprot (accessed on 28 December 2021).
