# Supplementary material for: Signal Transduction by VIP and PACAP Receptors
Source: Biomedicines. 2022 Feb 9;10(2):406. doi: 10.3390/biomedicines10020406 (PMC8962308; doi:10.3390/biomedicines10020406)
Supplement: Supplementary file 1 [file biomedicines-10-00406-s001.zip › biomedicines-1573471-supplementary.pdf]

Supplementary

Signal transduction by VIP and PACAP receptors

Ingrid Langer[1], Jérôme Jeandriens[1], Alain Couvineau[2], Swapnil Sanmukh[3], Dorota Latek[3,*]

[1]Institut de Recherche Interdisciplinaire en Biologie Humaine et Moléculaire (IRIBHM), Université libre de Bruxelles, B-1070 Brussels, Belgium

[2] Université de Paris, UMR 1149 Inserm - Centre de Recherche sur l'Inflammation (CRI), 75018 Paris, France

[3]Faculty of Chemistry, University of Warsaw, 02-093 Warsaw, Poland

Figure S1. *Network of all class B receptors and their peptide endogenous agonists.* This network of associations in Homo Sapiens was obtained from Szklarczyk et al. (the highest confidence level setting). Close associations between: VIP and GHRH; SCT and PACAP (ADCYAP1) and VIPR1; VIP and CALCA were detected.

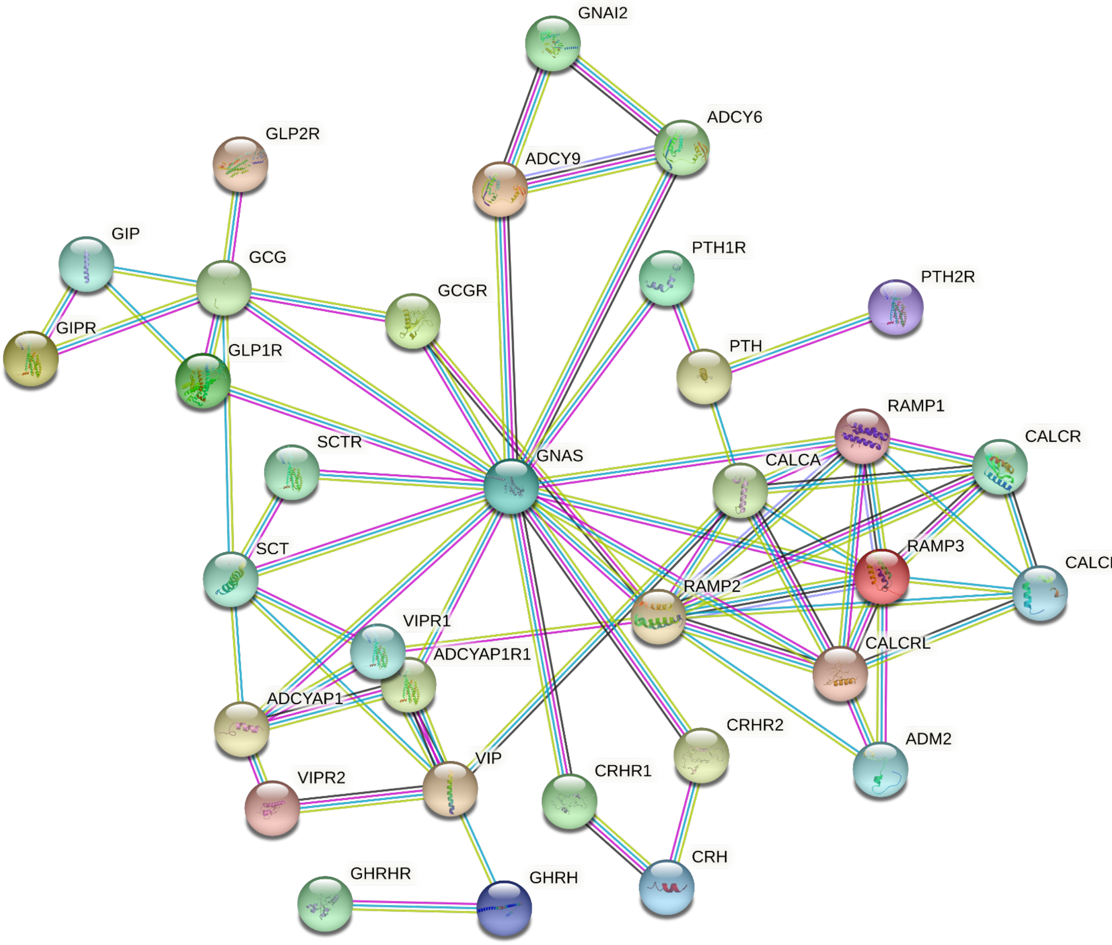

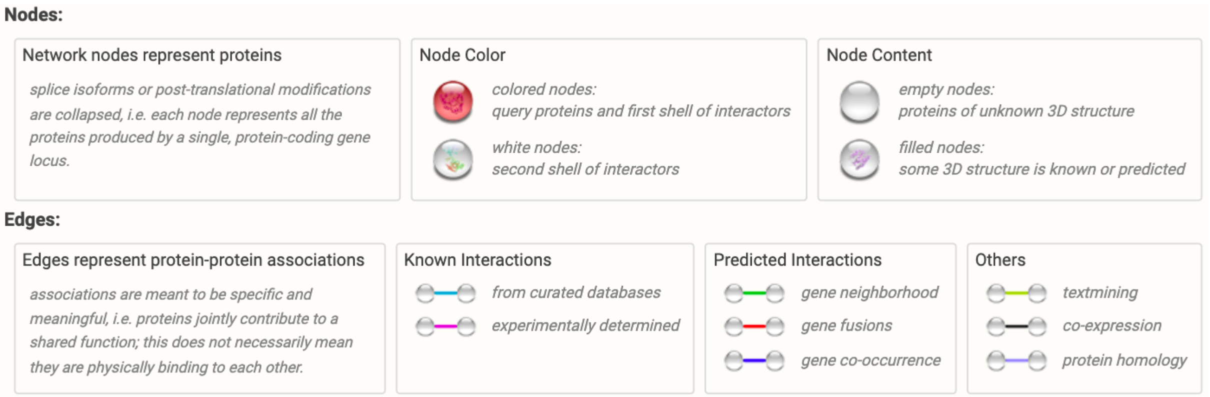

Figure S2. *Sequence variation in TM helices of class B GPCRs.* Amino acids were marked from the least conserved among class B GPCR sequences (blue) to the most conserved (red). The most conserved amino acids were marked in the right panel representing an inactive conformation of VPAC1 based on 5VEW.

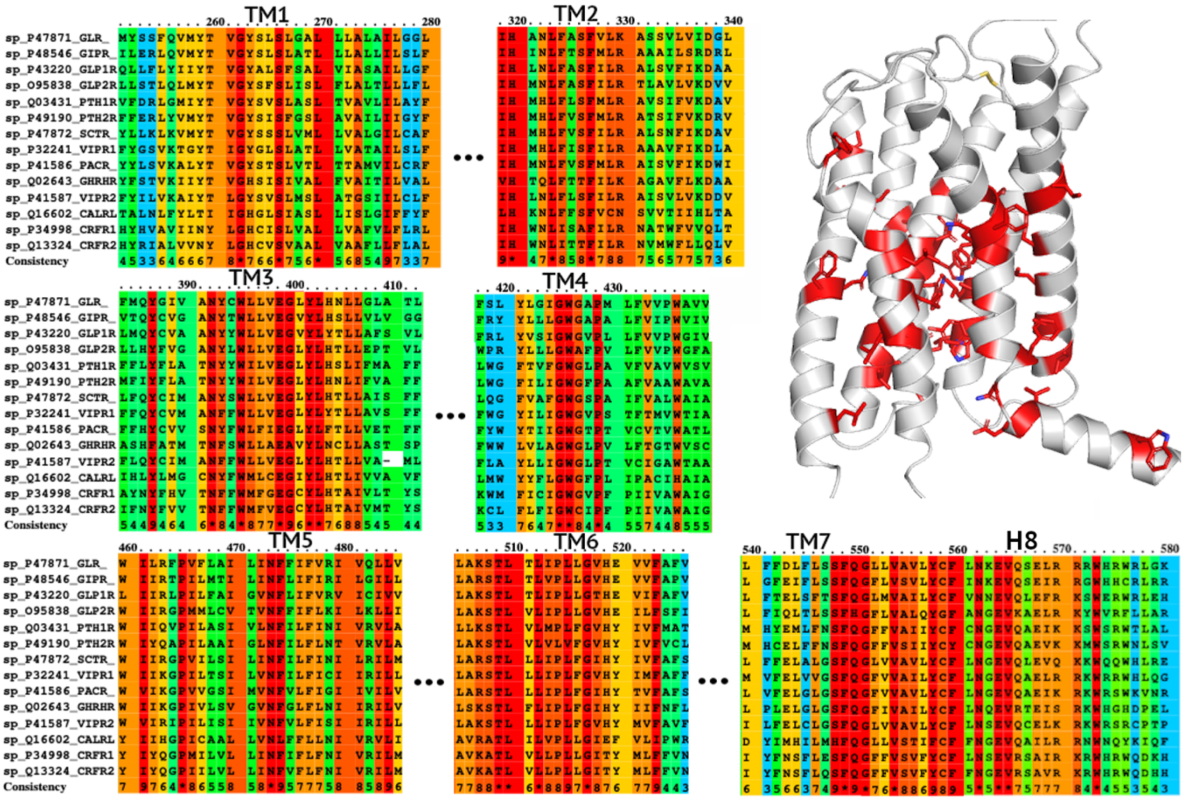

Figure S3. *Co-occurrence of class B GPCRs and their peptide agonists in various species obtained from Szklarczyk et al. 2021.*

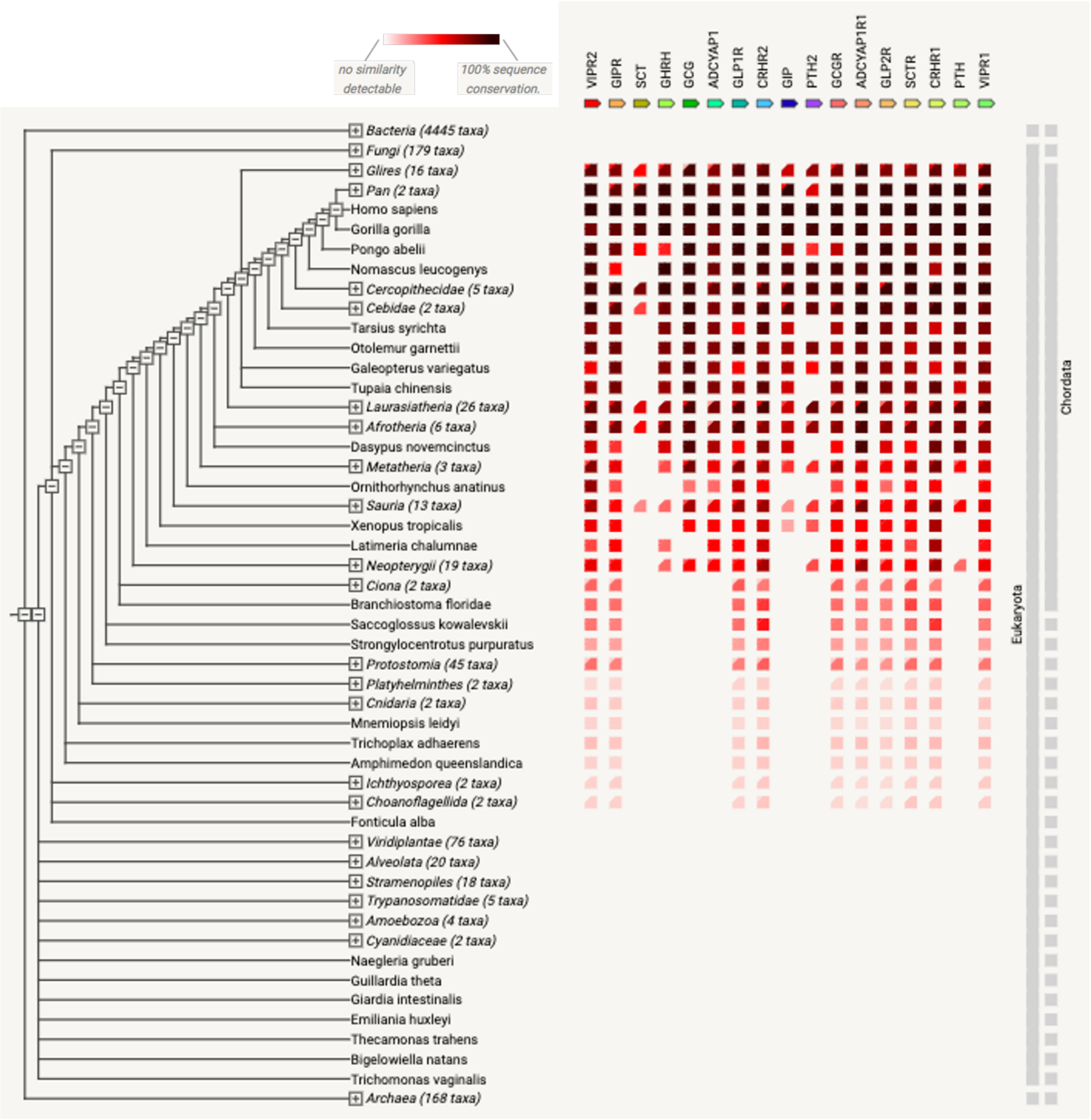

Figure S4. *Isoforms of VPAC1 deposited in NCBI.*

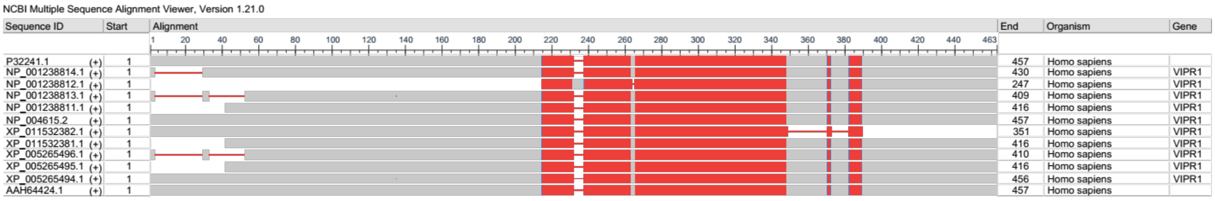

Figure S5. *Isoforms of VPAC2 deposited in NCBI.*

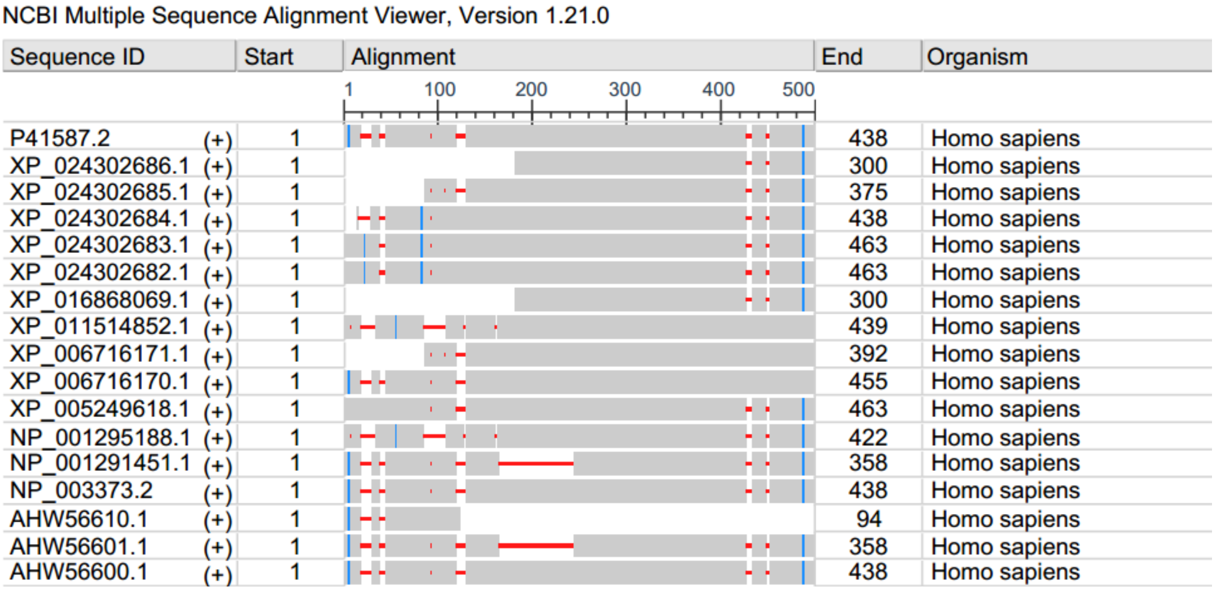

Figure S6. *Multiple sequence alignment (MSA) of five VPAC1 sequences deposited in Uniprot.*

```
P32241   VIPR1_HUMAN          1  MRPPSPLP-----ARWLCVLAG----ALAWAL-------GPA----G------------     27
P32241-2 VIPR1_HUMAN          1  MPPPPLLSLRRLGGGWSAVTRLVVAAAGARSRGGRGGSRGAGGGGRGGVARRRRLELRAA     60
P32241-3 VIPR1_HUMAN          1  ------------------------------------------------------------      0
P32241-4 VIPR1_HUMAN          1  ------------------------------------------------------------      0
P32241-5 VIPR1_HUMAN          1  ------------------------------------------------------------      0


P32241   VIPR1_HUMAN         28  --GQAARLQEECDYVQMIEVQHKQCLEEAQLENETIGCSKMWDNLTCWPATPRGQVVVLA     85
P32241-2 VIPR1_HUMAN         61  RSLLGSSLQEECDYVQMIEVQHKQCLEEAQLENETIGCSKMWDNLTCWPATPRGQVVVLA    120
P32241-3 VIPR1_HUMAN          1  ------------------------------------------------------------      0
P32241-4 VIPR1_HUMAN          1  ----------------------MRAGRRPRLGPWAGGCSKMWDNLTCWPATPRGQVVVLA     38
P32241-5 VIPR1_HUMAN          1  ----------------MIEVQHKQCLEEAQLENETIGCSKMWDNLTCWPATPRGQVVVLA     44


P32241   VIPR1_HUMAN         86  CPLIFKLFSSIQGRNVSRSCTDEGWTHLEPGPYPIACGLDDKAASLDEQQTMFYGSVKTG    145
P32241-2 VIPR1_HUMAN        121  CPLIFKLFSSIQGRNVSRSCTDEGWTHLEPGPYPIACGLDDKAASLDEQQTMFYGSVKTG    180
P32241-3 VIPR1_HUMAN          1  ------------------------------------------------------------      0
P32241-4 VIPR1_HUMAN         39  CPLIFKLFSSIQGRNVSRSCTDEGWTHLEPGPYPIACGLDDKAASLDE-QTMFYGSVKTG     97
P32241-5 VIPR1_HUMAN         45  CPLIFKLFSSIQGRNVSRSCTDEGWTHLEPGPYPIACGLDDKAASLDEQQTMFYGSVKTG    104


P32241   VIPR1_HUMAN        146  YTIGYGLSLATLLVATAILSLFRKLHCTRNYIHMHLFISFILRAAAVFIKDLALFDSGES    205
P32241-2 VIPR1_HUMAN        181  YTIGYGLSLATLLVATAILSLFRKLHCTRNYIHMHLFISFILRAAAVFIKDLALFDSGES    240
P32241-3 VIPR1_HUMAN          1  ------------------------------------------------------------      0
P32241-4 VIPR1_HUMAN         98  YTIGYGLSLATLLVATAILSLFRKLHCTRNYIHMHLFISFILRAAAVFIKDLALFDSGES    157
P32241-5 VIPR1_HUMAN        105  YTIGYGLSLATLLVATAILSLFRKLHCTRNYIHMHLFISFILRAAAVFIKDLALFDSGES    164


P32241   VIPR1_HUMAN        206  DQCSEGSVGCKAAMVFFQYCVM------ANFFWLLVEGLYLYTLLAVSFFSERKYFWGYI    259
P32241-2 VIPR1_HUMAN        241  DQCSEGSVGCKAAMVFFQYCVM------ANFFWLLVEGLYLYTLLAVSFFSERKYFWGYI    294
P32241-3 VIPR1_HUMAN          1  ---------MTRQRVWMRWAVRQPWSFSNIVSWLTSSGCWWRASTCTPCLPSPSSLS--G     49
P32241-4 VIPR1_HUMAN        158  DQCSEGSVGCKAAMVFFQYCVM------ANFFWLLVEGLYLYTLLAVSFFSERKYFWGYI    211
P32241-5 VIPR1_HUMAN        165  DQCSEGSVGCKAAMVFFQYCVM------ANFFWLLVEGLYLYTLLAVSFFSERKYFWGYI    218
                                          .   *::::.*         . **  .* :  :  ..  : . . :

P32241   VIPR1_HUMAN        260  LIGWGVPSTFTMVWTIARIHFEDYGCWDTINSSLWWIIKGPILTSILVNFILFICIIRIL    319
P32241-2 VIPR1_HUMAN        295  LIGWGVPSTFTMVWTIARIHFEDYGCWDTINSSLWWIIKGPILTSILVNFILFICIIRIL    354
P32241-3 VIPR1_HUMAN         50  STSGGVPSTFTMVWTIARIHFEDYGCWDTINSSLWWIIKGPILTSILVNFILFICIIRIL    109
P32241-4 VIPR1_HUMAN        212  LIGWGVPSTFTMVWTIARIHFEDYGCWDTINSSLWWIIKGPILTSILVNFILFICIIRIL    271
P32241-5 VIPR1_HUMAN        219  LIGWGVPSTFTMVWTIARIHFEDYGCWDTINSSLWWIIKGPILTSILVNFILFICIIRIL    278
                                  . ********************************************************

P32241   VIPR1_HUMAN        320  LQKLRPPDIRKSDSSPYSRLARSTLLLIPLFGVHYIMFAFFPDNFKPEVKMVFELVVGSF    379
P32241-2 VIPR1_HUMAN        355  LQKLRPPDIRKSDSSPYSRLARSTLLLIPLFGVHYIMFAFFPDNFKPEVKMVFELVVGSF    414
P32241-3 VIPR1_HUMAN        110  LQKLRPPDIRKSDSSPYSRLARSTLLLIPLFGVHYIMFAFFPDNFKPEVKMVFELVVGSF    169
P32241-4 VIPR1_HUMAN        272  LQKLRPPDIRKSDSSPYSRLARSTLLLIPLFGVHYIMFAFFPDNFKPEVKMVFELVVGSF    331
P32241-5 VIPR1_HUMAN        279  LQKLRPPDIRKSDSSPYSRLARSTLLLIPLFGVHYIMFAFFPDNFKPEVKMVFELVVGSF    338
                                ************************************************************

P32241   VIPR1_HUMAN        380  QGFVVAILYCFLNGEVQAELRRKWRRWHLQGVLGWNPKYRHPSGGSNGATCSTQVSMLTR    439
P32241-2 VIPR1_HUMAN        415  QGFVVAILYCFLNGEVQAELRRKWRRWHLQGVLGWNPKYRHPSGGSNGATCSTQVSMLTR    474
P32241-3 VIPR1_HUMAN        170  QGFVVAILYCFLNGEVQAELRRKWRRWHLQGVLGWNPKYRHPSGGSNGATCSTQVSMLTR    229
P32241-4 VIPR1_HUMAN        332  QGFVVAILYCFLNGEVQAELRRKWRRWHLQGVLGWNPKYRHPSGGSNGATCSTQVSMLTR    391
P32241-5 VIPR1_HUMAN        339  QGFVVAILYCFLNGEVQAELRRKWRRWHLQGVLGWNPKYRHPSGGSNGATCSTQVSMLTR    398
                                ************************************************************

P32241   VIPR1_HUMAN        440  VSPGARRSSSFQAEVSLV                                              457
P32241-2 VIPR1_HUMAN        475  VSPGARRSSSFQAEVSLV                                              492
P32241-3 VIPR1_HUMAN        230  VSPGARRSSSFQAEVSLV                                              247
P32241-4 VIPR1_HUMAN        392  VSPGARRSSSFQAEVSLV                                              409
P32241-5 VIPR1_HUMAN        399  VSPGARRSSSFQAEVSLV                                              416
                                ******************
```

Figure S7. *MSA of two VPAC2 sequences deposited in Uniprot.*

```
P41587   VIPR2_HUMAN          1  MRTLLPPALLTCWLLAPVNSIHPECRFHLEIQEEETKCAELLRSQTE------KHKACSG
P41587-2 VIPR2_HUMAN          1  ---------MPLWEAPSDHPANPPATLQG--HTSLPGCQEEPARDPQSGLPQITSESSSF
                                         :  *     :  :* . ::   : .   * *    : :      . ::.*

P41587   VIPR2_HUMAN         55  VWDNITCWRPANVGETVTVPCPKVFSNFYSKAGNISKNCTSDGWSETFPDFVDACGYSDP
P41587-2 VIPR2_HUMAN         50  SEGSLPSWSSGPAGAKLNASHE-----------GIGSSSDGNGDSKAATERVVSAMDTVR
                                  ..: .*  . .* .:..              .*.... .:* *::  : * :.  :

P41587   VIPR2_HUMAN        115  EDESKITFYILVKAIYTLGYSVSLMSLATGSIILCLFRKLHCTRNYIHLNLFLSFILRAI
P41587-2 VIPR2_HUMAN         99  RKHPEITFYILVKAIYTLGYSVSLMSLATGSIILCLFRKLHCTRNYIHLNLFLSFILRAI
                                ... :*******************************************************

P41587   VIPR2_HUMAN        175  SVLVKDDVLYSSSGTLHCPDQPSSWVGCKLSLVFLQYCIMANFFWLLVEGLYLHTLLVAM
P41587-2 VIPR2_HUMAN        159  SVLVKDDVLYSSSGTLHCPDQPSSWVGCKLSLVFLQYCIMANFFWLLVEGLYLHTLLVAM
                                ************************************************************

P41587   VIPR2_HUMAN        235  LPPRRCFLAYLLIGWGLPTVCIGAWTAARLYLEDTGCWDTNDHSVPWWVIRIPILISIIV
P41587-2 VIPR2_HUMAN        219  LPPRRCFLAYLLIGWGLPTVCIGAWTAARLYLEDTGCWDTNDHSVPWWVIRIPILISIIV
                                ************************************************************

P41587   VIPR2_HUMAN        295  NFVLFISIIRILLQKLTSPDVGGNDQSQYKRLAKSTLLLIPLFGVHYMVFAVFPISISSK
P41587-2 VIPR2_HUMAN        279  NFVLFISIIRILLQKLTSPDVGGNDQSQYKRLAKSTLLLIPLFGVHYMVFAVFPISISSK
                                ************************************************************

P41587   VIPR2_HUMAN        355  YQILFELCLGSFQGLVVAVLYCFLNSEVQCELKRKWRSRCPTPSASRDYRVCGSSFSRNG
P41587-2 VIPR2_HUMAN        339  YQILFELCLGSFQGLVVAVLYCFLNSEVQCELKRKWRSRCPTPSASRDYRVCGSSFSRNG
                                ************************************************************

P41587   VIPR2_HUMAN        415  SEGALQFHRGSRAQSFLQTETSVI
P41587-2 VIPR2_HUMAN        399  SEGALQFHRGSRAQSFLQTETSVI
                                ************************
```

Figure S8. *MSA of five PAC1 sequences deposited in Uniprot.*

```
P41586   PACR_HUMAN          1 MAGVVHVSLAALLLLPMAPAMHSDCIFKKEQAMCLEKIQRANELMGFNDSSPGCPGMWDN      60
P41586-2 PACR_HUMAN          1 MAGVVHVSLAALLLLPMAPAMHSDCIFKKEQAMCLEKIQRANELMGFNDSSPGCPGMWDN      60
P41586-3 PACR_HUMAN          1 MAGVVHVSLAALLLLPMAPAMHSDCIFKKEQAMCLEKIQRANELMGFNDSSPGCPGMWDN      60
P41586-4 PACR_HUMAN          1 MAGVVHVSLAALLLLPMAPAMHSDCIFKKEQAMCLEKIQRANELMGFNDSSPGCPGMWDN      60
P41586-5 PACR_HUMAN          1 MAGVVHVSLAALLLLPMAPAMHSDCIFKKEQAMCLEKIQRANELMGFNDSSP--------      52
                               ****************************************************

P41586   PACR_HUMAN         61 ITCWKPAHVGEMVLVSCPELFRIFNPDQVWETETIGESDFGDSNSLDLSDMGVVSRNCTE     120
P41586-2 PACR_HUMAN         61 ITCWKPAHVGEMVLVSCPELFRIFNPDQVWETETIGESDFGDSNSLDLSDMGVVSRNCTE     120
P41586-3 PACR_HUMAN         61 ITCWKPAHVGEMVLVSCPELFRIFNPDQ---------------------DMGVVSRNCTE      99
P41586-4 PACR_HUMAN         61 ITCWKPAHVGEMVLVSCPELFRIFNPDQ---------------------DMGVVSRNCTE      99
P41586-5 PACR_HUMAN         53 -------------------------------------------------DMGVVSRNCTE      63
                                                                                ***********

P41586   PACR_HUMAN        121 DGWSEPFPHYFDACGFDEYESETGDQDYYYLSVKALYTVGYSTSLVTLTTAMVILCRFRK     180
P41586-2 PACR_HUMAN        121 DGWSEPFPHYFDACGFDEYESETGDQDYYYLSVKALYTVGYSTSLVTLTTAMVILCRFRK     180
P41586-3 PACR_HUMAN        100 DGWSEPFPHYFDACGFDEYESETGDQDYYYLSVKALYTVGYSTSLVTLTTAMVILCRFRK     159
P41586-4 PACR_HUMAN        100 DGWSEPFPHYFDACGFDEYESETGDQDYYYLSVKALYTVGYSTSLVTLTTAMVILCRFRK     159
P41586-5 PACR_HUMAN         64 DGWSEPFPHYFDACGFDEYESETGDQDYYYLSVKALYTVGYSTSLVTLTTAMVILCRFRK     123
                               ************************************************************

P41586   PACR_HUMAN        181 LHCTRNFIHMNLFVSFMLRAISVFIKDWILYAEQDSNHCFISTVECKAVMVFFHYCVVSN     240
P41586-2 PACR_HUMAN        181 LHCTRNFIHMNLFVSFMLRAISVFIKDWILYAEQDSNHCFISTVECKAVMVFFHYCVVSN     240
P41586-3 PACR_HUMAN        160 LHCTRNFIHMNLFVSFMLRAISVFIKDWILYAEQDSNHCFISTVECKAVMVFFHYCVVSN     219
P41586-4 PACR_HUMAN        160 LHCTRNFIHMNLFVSFMLRAISVFIKDWILYAEQDSNHCFISTVECKAVMVFFHYCVVSN     219
P41586-5 PACR_HUMAN        124 LHCTRNFIHMNLFVSFMLRAISVFIKDWILYAEQDSNHCFISTVECKAVMVFFHYCVVSN     183
                               ************************************************************

P41586   PACR_HUMAN        241 YFWLFIEGLYLFTLLVETFFPERRYFYWYTIIGWGTPTVCVTVWATLRLYFDDTGCWDMN     300
P41586-2 PACR_HUMAN        241 YFWLFIEGLYLFTLLVETFFPERRYFYWYTIIGWGTPTVCVTVWATLRLYFDDTGCWDMN     300
P41586-3 PACR_HUMAN        220 YFWLFIEGLYLFTLLVETFFPERRYFYWYTIIGWGTPTVCVTVWATLRLYFDDTGCWDMN     279
P41586-4 PACR_HUMAN        220 YFWLFIEGLYLFTLLVETFFPERRYFYWYTIIGWGTPTVCVTVWATLRLYFDDTGCWDMN     279
P41586-5 PACR_HUMAN        184 YFWLFIEGLYLFTLLVETFFPERRYFYWYTIIGWGTPTVCVTVWATLRLYFDDTGCWDMN     243
                               ************************************************************

P41586   PACR_HUMAN        301 DSTALWWVIKGPVVGSIMVNFVLFIGIIVILVQKLQSPDMGGNESSIY------------     348
P41586-2 PACR_HUMAN        301 DSTALWWVIKGPVVGSIMVNFVLFIGIIVILVQKLQSPDMGGNESSIYFSCVQKCYCKPQ     360
P41586-3 PACR_HUMAN        280 DSTALWWVIKGPVVGSIMVNFVLFIGIIVILVQKLQSPDMGGNESSIY------------     327
P41586-4 PACR_HUMAN        280 DSTALWWVIKGPVVGSIMVNFVLFIGIIVILVQKLQSPDMGGNESSIYFSCVQKCYCKPQ     339
P41586-5 PACR_HUMAN        244 DSTALWWVIKGPVVGSIMVNFVLFIGIIVILVQKLQSPDMGGNESSIY------------     291
                               ************************************************

P41586   PACR_HUMAN        349 ----------------LRLARSTLLLIPLFGIHYTVFAFSPENVSKRERLVFELGLGSFQ     392
P41586-2 PACR_HUMAN        361 RAQQHSCKMSELSTITLRLARSTLLLIPLFGIHYTVFAFSPENVSKRERLVFELGLGSFQ     420
P41586-3 PACR_HUMAN        328 ----------------LRLARSTLLLIPLFGIHYTVFAFSPENVSKRERLVFELGLGSFQ     371
P41586-4 PACR_HUMAN        340 RAQQHSCKMSELSTITLRLARSTLLLIPLFGIHYTVFAFSPENVSKRERLVFELGLGSFQ     399
P41586-5 PACR_HUMAN        292 ----------------LRLARSTLLLIPLFGIHYTVFAFSPENVSKRERLVFELGLGSFQ     335
                                               ********************************************

P41586   PACR_HUMAN        393 GFVVAVLYCFLNGEVQAEIKRKWRSWKVNRYFAVDFKHRHPSLASSGVNGGTQLSILSKS     452
P41586-2 PACR_HUMAN        421 GFVVAVLYCFLNGEVQAEIKRKWRSWKVNRYFAVDFKHRHPSLASSGVNGGTQLSILSKS     480
P41586-3 PACR_HUMAN        372 GFVVAVLYCFLNGEVQAEIKRKWRSWKVNRYFAVDFKHRHPSLASSGVNGGTQLSILSKS     431
P41586-4 PACR_HUMAN        400 GFVVAVLYCFLNGEVQAEIKRKWRSWKVNRYFAVDFKHRHPSLASSGVNGGTQLSILSKS     459
P41586-5 PACR_HUMAN        336 GFVVAVLYCFLNGEVQAEIKRKWRSWKVNRYFAVDFKHRHPSLASSGVNGGTQLSILSKS     395
                               ************************************************************

P41586   PACR_HUMAN        453 SSQIRMSGLPADNLAT                                                 468
P41586-2 PACR_HUMAN        481 SSQIRMSGLPADNLAT                                                 496
P41586-3 PACR_HUMAN        432 SSQIRMSGLPADNLAT                                                 447
P41586-4 PACR_HUMAN        460 SSQIRMSGLPADNLAT                                                 475
P41586-5 PACR_HUMAN        396 SSQIRMSGLPADNLAT                                                 411
                               ****************
```

Table S1. *Major isoforms of VIP and PACAP receptors according to Uniprot (accession date: 28 Dec 2021).*

Receptor	Accession number	Name	Sequence length	Sequence modifications with respect to the reference sequence (isoform 1)
**VPAC1**	P32241-1	reference sequence, isoform 1	457	
	P32241-2	Isoform 2	492	Additional fragment in N-termini
	P32241-3	Isoform 3	247	5TM sequence
	P32241-4	Isoform 4	409	Short variant according to Ota et al.
	P32241-5	Isoform 5	416	Short variant according to Ota et al.
**VPAC2**	P41587-1	Reference sequence, isoform 1	438	
	P41587-2	Isoform 2	422	Short variant according to Ota et al.
**PAC1**	P41586-1	Reference sequence, PAC1 null, isoform N, isoform 1	468	
	P41586-2	PAC1 hop, Isoform N-HOP1	496	insertion hop cassette
	P41586-3	PAC1 δ5-6 null, Isoform S	447	deletion exons 5-6
	P41586-4	PAC1 δ5-6 hop, Isoform S-HOP1	475	insertion hop cassette + deletion exons 5-6
	P41586-5	PAC1 δ4-6 null, Isoform VS	411	deletion exons 4-6
	NCBI ref XP_006715708.1	PAC1 hip, Isoform X1	496	insertion hip cassette
